# Prevalence and factors associated with early initiation of breastfeeding among Bangladeshi mothers: A nationwide cross-sectional study

**DOI:** 10.1371/journal.pone.0215733

**Published:** 2019-04-25

**Authors:** Md. Ariful Islam, ASMA Mamun, Md. Murad Hossain, Premananda Bharati, Aik Saw, Pete E. Lestrel, Md. Golam Hossain

**Affiliations:** 1 Department of Statistics, University of Rajshahi, Rajshahi, Bangladesh; 2 Department of Biotechnology and Genetic Engineering, Faculty of Science, Noakhali Science and Technology University, Noakhali, Bangladesh; 3 Biological Anthropology Unit, Indian Statistical Institute, Kolkata, West Bengal, India; 4 National Orthopaedic Centre of Excellence for Research and Learning (NOCERAL), Department of Orthopaedic Surgery, University of Malaya, Kuala Lumpur, Malaysia; 5 Sections of Orthodontics and Oral Biology, School of Dentistry, University of California, Los Angeles, California, United States of America; University of South Carolina Arnold School of Public Health, UNITED STATES

## Abstract

**Background:**

Early initiation of breastfeeding (EIBF) is associated with better health of the mothers and reduced risk of neonatal mortality. The objective of this study was to determine the prevalence of EIBF and associated factors among Bangladeshi mothers.

**Methods:**

The data was extracted from the Bangladesh Demographic and Health Survey (BDHS)-2014. A total of 4,092 married non-pregnant Bangladeshi mothers who had at least one child aged 2 years or younger were included in this study. A two-level logistic regression model was used to remove the clustering effect for finding the impact of socio-economic and demographic factors on EIBF.

**Results:**

The prevalence of EIBF among Bangladeshi mothers was 51.4% (urban: 47.1% and rural: 53.4%). A two -level logistic regression model showed that mothers living in the Sylhet division (p<0.01) and rural environment (p<0.05) were more likely to practice EIBF. Mothers who were obese or overweight (p<0.01), had secondary (p<0.05) or higher education (p<0.01) were less likely to provide early breastfeeding to their newborn babies compared to their counterparts. Those who delivered by caesarian-section (p<0.01) were less likely to perform EIBF while those who attended an antenatal care clinic more than 3 times (p<0.05) were more likely to do so.

**Conclusions:**

About half of the Bangladeshi mothers did not start breast-feeding within one hour after birth. This study identified several geographical and socio-demographic factors that were associated with EIBF, and hope that this information will help the government to focus their resources to promote early breastfeeding.

## Introduction

The World Health Organization (WHO) considers breastfeeding as the normal way to provide young infants with all the nutrients that they need for healthy growth and development especially in the first six months of life [1]. It has been shown that breast milk contains essential nutrients with immunological and anti-inflammatory properties that protect both mothers and children against various infections and diseases [2–4]. Early initiation of breastfeeding (EIBF) or timely initiation of breastfeeding refers to providing breast milk to the newborn babies within one hour of birth, which ensures that they receive essential nutrients including the colostrums [5]. Colostrum, the “first milk” produced by the mother first few days after delivery is endowed with protective antibodies that act as the first line of defense in the form of passive immunization for the infant. The colostrum fortifies their immune system and contributes towards the lowering of the mortality rate in neonates [6]. It also contains at least ninety known nutritional components including amino acids, minerals and vitamins essential for the growth and development of newborns [7]. EIBF increases skin-to-skin contact between mother and infant, and helps to prevent hypothermia of the baby, establishes bonding between mother and child, and increases the potential for exclusive breastfeeding practices [8].

Late initiation of breastfeeding has been associated with a fivefold increase in morbidity and mortality due to conditions such as diarrheal diseases [9]. Infectious diseases and malnutrition are also major causes of death among infants in developing countries [9, 10]. Researchers have reported that children who received initial breastfeeding were at a lower risk of having acute respiratory and gastrointestinal infections compared to children who did not [11]. Effective implementation of EIBF has been reported to reduce the death rate 13%-15% among children less than 5 years of age among middle and low-income communities in Tanzania [12]. Moreover, EIBF has also been shown to reduce the risk of postpartum hemorrhage, which is one of the leading causes of maternal mortality [6]. Prevalence of EIBF reflects the practice of placing newborn children on the breast within one hour of birth [13]. Therefore, it is hoped that the practice of EIBF will gradually gain worldwide attention [14–22].

In Bangladesh, the trend of practicing EIBF among lactating mothers has remained mostly unchanged for a long time [23]. Recent data on under-five child mortality rates was 46 per 1000 live births, and of which 61% were contributed by neonatal mortality [23]. EIBF reduces the risk for all causes of mortality in neonates including babies with low birth weight. EIBF was found to reduce 22% of neonatal deaths and 16% of all infant deaths [24]. More recently it was found that mothers that did not breastfeed their newborn within one hour after their birth, the odds of neonatal deaths were increased by nearly threefold in comparison with those neonates who were breastfed within one hour of birth in India [25]. The government of Bangladesh is currently working towards achieving the target for Sustainable Development Goals (SDGs) by 2030, and one of the important components is to reduce the maternal and under-five child mortality rates to less than 70 per 100,000 live births and less than 25 per 1000 live births respectively [26]. Since there is a strong association between EIBF and the good health of newborns as well as children and their mothers, information on the pattern of breastfeeding practices at a national level would be very useful for the health authorities to introduce or enhance EIBF among mothers nationwide.

The objective of this study is then to determine the prevalence of EIBF and investigate its association with socio-economic and demographic factors among Bangladeshi mothers.

## Methods

The 2014 BDHS received ethics approval from Bangladesh Medical Research Council. The 2014 BDHS also received written consent from each individual in the study. The data used in the present study was extracted from the large scale of dataset collected by the Bangladesh Demographic and Health Survey (BDHS-2014). This is a nationally representative survey, which covered all administrative regions (or divisions) of Bangladesh. Thus, Bangladesh is divided into eight administrative divisions, of which Mymensinghm is a new division. During the BDHS-2014, Bangladesh was divided into seven administrative divisions, and Mymensinghm was considered as a district under the Dhaka division. The BDHS-2014 survey collected socio-demographic, health, anthropometric and lifestyle information from 17,863 Bangladeshi married women over the reproductive age (15 to 49 years). The data was collected from March 24, 2014 to August 11, 2014. The sampling technique, survey design, survey instruments, measuring system and quality control have been described elsewhere [23].

### Sampling

BDHS-2014 utilized a two-stage stratified cluster sampling procedure for selecting the sample from the Bangladesh survey. In the first stage, 600 enumeration areas (EAs) (207 from urban and 393 from rural areas) were randomly selected with a probability proportional to the size of the EAs. In the second stage, 30 households were selected from each EA using systematic sampling, which covered the whole country. Using this design, 18,000 residential households were selected for interviews of 18,000 ever-married women. BDHS-2014 collected data on infant feeding with the youngest children under the age 2 who were living with their mothers, using a 24-hour recall method [23].

**Fig 1:**
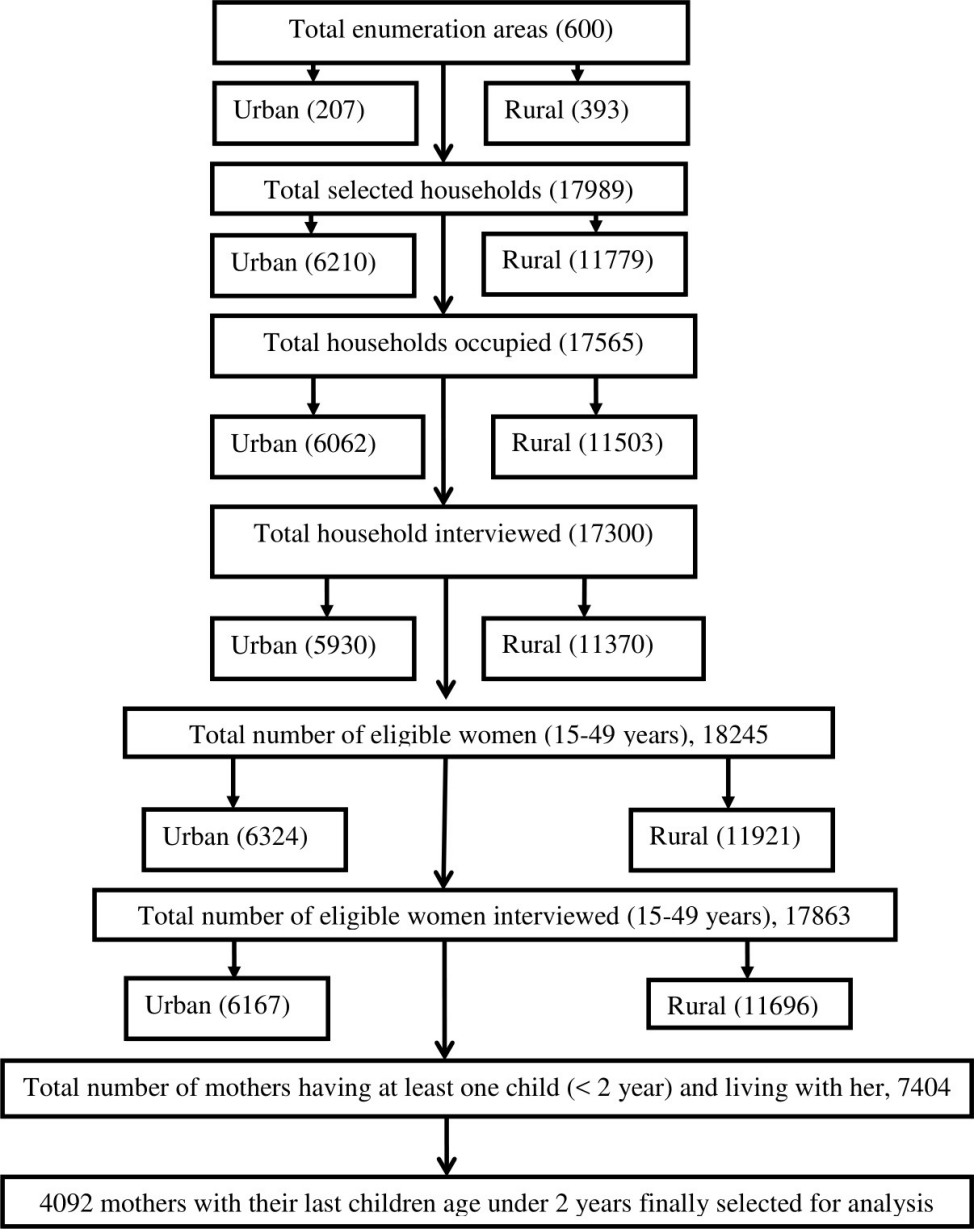
Sample selection procedure for analysis.

Abnormal data points were then identified and excluded because their presence may lead to misleading results [27]. Some missing values were also detected, and these cases were also excluded. Pregnant and women who did not have children of age below 2 years old were also excluded from the study. After these adjustments, the data set was reduced to 4,092 mothers for further analysis (Fig 1).

### Outcome variable

EIBF was considered the outcome variable in this study. The BDHS-2014 study asked the following question of the mothers: how long after birth did you first put (name) to the breast? According to the WHO, initiation of breastfeeding was expressed as a dichotomous variable with category 1 for initiation of breastfeeding within one hour (early initiation) and category 0 for initiation of breastfeeding after one hour (late initiation).

### Independent variables

A number of independent variables were selected with reference to an earlier study on the breastfeeding practice conducted in Nepal [28]. The following independent variables were used: geographical location (division) (Dhaka, 1; Chittagong, 2; Barisal, 3; Khulna, 4; Rajshahi, 5; Rangpur, 6; Sylhet, 7), type of residence (urban, 1; rural, 2), religion (Muslim, 1; Non-Muslim, 2), mothers, education level (uneducated, 0; primary, 1; secondary, 2; higher, 3), wealth index (this is a composite index, and it was calculated using some selected assets data. It was constructed by principal components analysis) (poor, 1; middle, 2; rich, 3), currently working employment of the mother (not working, 0; currently working, 1), age of mother (age ≤20 years, 1; 21–29 years, 2; age ≥30years, 3), age at first marriage (≥18 years, 0; <18 years,1), age at first child birth (age ≥20years, 0; age <20years, 1), marital status (currently married, 0; single, 1), number of ever born children (1–2 children, 1; 3–5 children, 2; 6 and more children, 3), child’s birth weight (large, 1; average, 2; low, 3) (birth weight of children was not measured by BDHS-2014, they considered mother’s perception of the baby’s birth size), place of delivery (home, 0; hospital/clinic, 1), sex of last children (male, 1; female, 2), mode of delivery (caesarean, 1; vaginal, 0), marriage to first birth interval (within one year, 1; 2–3 years, 2; 4 and above years, 3), number of family members (≤4 member, 1; 5–10 member, 2; ≥11 member, 3), number of antenatal care visits (no, 0; 1–2 times, 1; 3 and more times, 2), mothers’ body mass index (BMI) category (normal weight1; underweight, 2; overweight, 3 and obese, 4).

## Statistical analysis

A frequency distribution (percentage) was used to determine the prevalence of EIBF among Bangladeshi mothers. Chi-square (χ^2^) tests were performed to evaluate the association between independent variables and the EIBF. BDHS-2014 collected data using two-stage stratified cluster sampling. Since the observations were derived from several levels of hierarchy, it was possible to obtain a clustering effect in outcome variable. A single-level statistical model would not be appropriate for analyzing this type of data set [29]. To remove the clustering effect, two levels of multiple logistic regression analysis were used to detect the impact of socioeconomic, demographic and nutritional factors on EIBF among Bangladeshi mothers. The median odds ratio (MOR) was used to check the existence of clustering effect in outcome variable. The MOR is defined as

$$\mathrm{MOR}=\exp\left\{0.6745\sqrt{2}\sqrt{\sigma_{u}^{2}}\right\}=\exp\left(0.95\sqrt{\sigma_{u}^{2}}\right),$$ (1)

where $\sigma_{u}^{2}$ is the cluster variance. The value of MOR is always greater than or equal to 1. If MOR = 1, it means there is no cluster variation, but if MOR>1, there is cluster variation of the outcome variable, and the effect needs to be removed [30].

A multilevel model is particularly appropriate for research designs where the data for participants are organized at more than one level. In this study, level I was considered for the individual level and level II for clusters (EAs).

The underlying two-level logistic regression model corresponding to each variable is:

$$\mathrm{Level}\;I:\eta_{ij}=\beta_{0j}+\beta_{1}x_{ij},P_{ij}=\frac{\exp(\eta_{ij})}{1+\exp(\eta_{ij})},\;\mathrm{where}\;y_{ij}=1$$ (2)

with probability *P*_*ij*_, *y*_*ij*_ = 0, with probability 1−*P*_*ij*_, in $\frac{P_{ij}}{1-P_{ij}}=\beta_{0j}+\beta_{1}x_{ij}$

$$\mathrm{Level}\;\mathrm{II}:\beta_{0j}=y_{00}+u_{0j},\beta_{1j}=y_{10},u_{0j}∼N\left(0,\tau_{00}\right),$$

$$\pi =p\left(Y|X_{1}=x_{1},X_{2}=x_{2},\ldots ,X_{p}=x_{p}\right)=\frac{\exp\left\{g(x_{i})\right\}}{1+\exp\left\{g(x_{i})\right\}},$$ (3)

where *g*(*x*_1_) = *β*_0_+*β*_1_*x*_*i*1_ +…+*β*_*k*_*x*_*ik*_;*i* = 1,2,…,*n*.

Where, *β*_*i*_ = unknown logistic regression coefficients (i = 1, 2, …, n). The parameter *β*_*i*_ refers to the effect of X_i_ on the log odds such that Y = 1, controlling the other X_i_. The multicollinearity problem among the independent variables was checked by standard error (SE). If the magnitude value of SE was less than 0.5 it was considered as no evidence of the multicollinearity problem [31]. BDHS-2014 did not use a proportional allocation of the samples with the national administrative divisions or with respect to their type of residence. Sampling weight was necessary for any statistical analysis using the BDHS-2014 data to ensure actual national representation of the survey results. In this paper sampling weights was calculated for univariate, bivariate and multivariate analysis. Statistical significance was accepted at p < 0.05. All statistical analyses were carried out using STATA (version 13) and SPSS software (IBM version 20).

## Results

### Prevalence of early initiation of breastfeeding (EIBF)

A total of 4,092 mothers with their last child under aged 2 were included in the study. The prevalence of EIBF among Bangladeshi mothers was 51.4%. Of the mothers, 53.4% came from a rural environment (67.4% of total sample population) and breastfeed their babies within one hour of birth.

### Association between EIBF and other factors

It was noted that the rate of EIBF among mothers in the rural areas of Bangladesh (53.4%) was significantly (p<0.01) higher than those living in the urban areas (47.1%). The association between location (division) and EIBF was also statistically significant (p<0.01), and it was found that the highest and lowest rate of EIBF were in Sylhet (59.6%) and Khulna (44.0%) divisions respectively. Among the mothers, 13.2% were uneducated, while 11.9% had a higher level of education. It was noted that the rate of EIBF significantly decreased (p < 0.01) with increasing education level of the mothers. A similar pattern was observed where mothers that came from rich families showed a significantly lower rate (p <0.01) of EIBF (47.0%) when compared to those from poor (54.9%) and middle (53.5%) income families. About two thirds (72%) of the mothers were married before 18 years of age. The rate of EIBF was significantly (p<0.05) higher (52.5%) among those who married before 18 years as compared to mothers who were married after 18 years (48.5%). About two thirds (72%) of the mothers were below 20 years of age when they delivered their first baby. It was noted that a significantly (p<0.01) higher rate of EIBF was observed among these early childbearing (53.0%) mothers when compared to those who were older than 20 years (46.9%) when they delivered their first child. Approximately 60% of the mothers delivered their last baby at home, and among them more than half (58.8%) practiced EIBF. This rate was significantly higher (p<0.01) than the rate of EIBF among mothers who delivered in a hospital/clinic. About one fourth of the mothers (24.5%) were delivered by caesarean section in Bangladesh. A significantly (p<0.01) higher rate of EIBF (58.3%) was noted among mothers who underwent vaginal delivery compared to those who delivered by caesarean section (30.1%). It was also observed that the rate of EIBF significantly (p<0.01) decreased with increasing body size among mothers in Bangladesh. Mothers who never attended any antenatal visits were significantly (p<0.05) less likely to practice EIBF (49.2%) compared to those who attended three or more antenatal visits (55.2%) (Table 1).

**Table 1:** Characteristics of the study participants by: 1) Early initiation of breastfeeding and 2) Prevalence of early initiation of breastfeeding. Bangladesh Demographic and Health Survey (BDHS), 2014 (N = 4092).

	Total	EIBF	Not EIBF	Prevalence of EIBF	χ^2^-value
Overall	4092	2102 (51.370)	1990 (48.630)	51.370	
Variables	n (%)	n (%)	n(%)	%	
**Type of residence**					14.340**
Urban	1333(32.600)	628 (47.100)	705 (52.900)	47.110	
Rural	2759(67.400)	1474 (53.400)	1285 (46.600)	53.430	
**Currently working**					3.433
No	3207(78.400)	1623(50.600)	1584 (49.400)	50.600	
Yes	885(21.600)	479(54.100)	406 (45.900)	54.120	
**Division**					49.554**
Dhaka	728(17.800)	355(48.800)	373 (51.200)	48.800	
Chittagong	779(19.000)	357 (45.800)	422 (54.200)	45.800	
Barisal	497(12.100)	266 (53.500)	231 (46.500)	53.500	
Khulna	493(12.000)	217(44.000)	276 (56.000)	44.000	
Rajshahi	504(12.300)	262 (52.000)	242 (48.000)	52.000	
Rangpur	527(12.900)	309 (58.600)	218 (41.400)	58.600	
Sylhet	564(13.800)	336(59.600)	228 (40.400)	59.600	
**Religion**					1.072
Muslim	3763(92.000)	1942 (51.600)	1821 (48.400)	51.600	
Non-Muslim	329(8.000)	160(48.600)	169 (51.400)	48.600	
**Age at first marriage**					5.335*
Age≥18 years	1141(27.900)	553(48.500)	558 (51.500)	48.500	
Age<18 years	2951(72.100)	1549 (52.500)	1402 (47.500)	52.500	
**Age at first birth**					11.974**
Age≥20 years	1112(27.200)	522(46.900)	590 (53.100)	46.900	
Age<20 years	2980(72.800)	1580(53.000)	1400 (47.000)	53.000	
**Marital Status**					2.895
Currently married	3450(84.300)	1792(51.900)	1658 (48.100)	51.900	
Single	642(15.700)	310(48.300)	332 (51.700)	48.300	
**No. of ever born children**					
1–2	2885(70.500)	1461(50.600)	1424 (49.400)	50.600	2.716
3–5	1074(26.200)	566(52.700)	508 (47.300)	52.700	
6 and more	133(3.3000)	75(56.400%)	58(43.600%)	56.400	
**Mothers’ education level**					32.687**
Uneducated	541(13.200)	312(57.700)	229 (42.300)	57.700	
Primary	1098(26.800)	593(54.000)	505 (46.000)	54.000	
Secondary	1966(48.000)	997(50.700)	969 (49.300)	50.700	
Higher	487(11.900)	200(41.100)	287 (58.900)	41.100	
**Child birth weight**					0.199
Large	542(13.200)	276(50.900)	266 (49.100)	50.900	
Average	2768(67.600)	1419(51.300)	1349 (48.700)	51.300	
Low	782(19.100)	407(52.000)	375 (48.000)	52.000	
**Wealth Index**					21.985**
Poor	1611(39.400)	884(54.900)	727 (45.100)	54.900	
Middle	797(19.500)	426(53.500)	371 (46.500)	53.500	
Rich	1684(41.200)	792(47.000)	892 (53.000)	47.000	
**Place of delivery**					130.286**
Home	2425(59.300)	1425(58.800)	1000 (41.200)	58.800	
Hospital/Clinic	1667(40.700)	677(40.600)	990 (59.400)	40.600	
**Sex of children**					2.987
Male	2127(52.000)	1065(50.100)	1062 (49.900)	50.100	
Female	1965(48.000)	1037(52.800)	928 (47.200)	52.800	
**Age group**					2.658
Age≤20 years	1143(27.900)	609(53.300)	534 (46.700)	53.300	
Age, 21–29 years	2151(52.600)	1082(50.300)	1069 (49.700)	50.300	
Age≥30	798(19.500)	411(51.500)	387 (48.500)	51.500	
**Marriage to first birth interval**					3.287
Within 1 year	1982(48.400)	1047(52.800)	935 (47.200)	52.800	
2–3 years	1509(36.900)	753(49.900)	756 (50.100)	49.900	
4 and above years	601(14.700)	302(50.200)	299(49.800)	50.200	
**Mode of delivery**					240.372**
Caesarean	1003(24.500)	302(30.100)	701(69.900)	30.100	
Vaginal	3089(75.500)	1800(58.300)	1289 (41.700)	58.300	
**No. of family member**					3.117
≤4	1248(30.500)	648(51.900)	600 (48.100)	51.900	
5–10	2569(62.800)	1300(50.600)	1269 (49.400)	50.600	
11 and more	275(6.700)	154(56.000)	121 (44.000)	56.000	
**No. of antenatal care visit**					8.155*
No	1901(46.500)	936(49.200)	965 (50.800)	49.200	
1–2 times	1347(32.900)	702(52.100)	645 (47.900)	52.100	
3 and more times	844(20.600)	464(55.000)	380 (45.000)	55.000	
**Mothers’ BMI category**					18.867**
Underweight	1036(25.300)	562(54.200)	474 (45.800)	54.200	
Normal weight	2369(57.900)	1234(52.100)	1135 (47.900)	52.100	
Over weight	577(14.100)	264(45.800)	313 (54.200)	45.800	
Obese	110(2.700)	42(38.200)	68 (61.800)	38.200	

N.B.: EIBF, early initiation of breastfeeding; χ^2^, Chi-square; **, p<0.01; and *, p<0.05.

### Effect of socio-economic and demographic factors on EIBF

This study found that the value of MOR was 1.596, suggesting the presence of clustering variation in the outcome variable among enumeration (clusters). Two-level logistic regression model was found to be an appropriate approach to analyze the data by removing the clustering effect to ensure a higher accuracy of the results. All the factors that demonstrated a significant association with the rate of EIBF were included as independent variables in the two-level logistic regression model. After removing clustering effects, and controlling for the effect of other variables, the model showed that mothers living in Dhaka division were less likely to initiate early breastfeeding than those living in Sylhet (AOR: 1.474; 95% CI: 1.118–1.944; p<0.01) and Rangpur (AOR: 1.479; 95% CI: 1.121–1.952; p<0.01) divisions. It was also found that rural mothers (AOR: 1.164; 95% CI: 0.977–0.972; p<0.05) were more likely to initiate breastfeeding early as compared to their urban counterparts. Education had a negative influence on EIBF, implying that mothers with secondary education (AOR: 0.814; 95% CI: 0.656–0.985; p<0.05) and higher education (AOR: 0.584; 95% CI: 0.439–0.779; p<0.01) were less likely to practice EIBF than those who were uneducated. The rate of EIBF among healthy (normal weight) mothers was 0.776 times higher (AOR: 0.776; CI: 0.646–0.931; p<0.001) compared to that of overweight mothers, and 0.568 times (AOR: 0.568; CI: 0.383–0.842; p<0.001) compared to that of obese mothers. Frequency of antenatal visits (ANC) was found to be significantly associated with EIBF among Bangladeshi mothers. Mothers who regularly attended the antenatal clinic (3 and more times) were 1.24 times more likely to practice EIBF compared to mothers who did not attend the ANC visits (AOR: 1.243; 95% CI: 1.024–1.510; p<0.05). It was also noted that mothers who underwent natural/vaginal delivery were more likely to provide initial breastfeeding earlier than mothers who underwent caesarean delivery (AOR: 0.302; 95% CI: 0.242–0.377; p<0.01) (Table 2).

**Table 2:** Effect of socio-economic and demographic factors on early initiation of breastfeeding among non-pregnant mothers in Bangladesh.

Variable	B	SE	z	p-value	AOR	95% CI for AOR	
						Lower	Upper
**Division**							
Chittagong Vs Dhaka^R^	-0.144	0.110	-1.130	0.259	0.865	0.673	1.112
Barisal Vs Dhaka^R^	0.181	0.170	1.250	0.210	1.199	0.902	1.593
Khulna Vs Dhaka^R^	-0.213	0.110	-1.500	0.133	0.807	0.610	1.067
Rajshahi Vs Dhaka^R^	0.126	0.160	0.900	0.369	1.135	0.860	1.497
Rangpur Vs Dhaka^R^	0.391	0.210	2.770	0.006	1.479	1.121	1.952
Sylhet Vs Dhaka^R^	0.388	0.210	2.750	0.006	1.474	1.118	1.944
**Type of Residence**							
Rural Vs Urban^R^	0.152	0.100	1.710	0.042	1.164	0.977	0.972
**Mother education level**							
Primary Vs Uneducated^R^	-0.138	0.100	-1.230	0.219	0.870	0.697	1.086
Secondary Vs Uneducated^R^	-0.204	0.100	-1.850	0.049	0.814	0.656	0.985
Higher Vs Uneducated^R^	-0.536	0.090	-3.670	0.001	0.584	0.439	0.779
**Place of delivery**							
Hospital/Clinic Vs Home^R^	-0.149	0.080	-1.520	0.127	0.861	0.711	1.043
**Mothers’ BMI**							
Underweight Vs Normal weight ^R^	0.087	0.080	0.770	0.246	1.091	0.942	1.262
Overweight Vs Normal weight ^R^	-0.254	0.090	-2.370	0.006	0.776	0.646	0.931
Obese Vs Normal weight ^R^	-0.565	0.200	-2.070	0.005	0.568	0.383	0.842
**Age at first marriage**							
<18 years Vs ≥18 years^R^	-0.082	0.090	-0.870	0.386	0.920	0.763	1.110
**Wealth index**							
Middle Vs Poor^R^	0.083	0.100	0.870	0.387	1.086	0.899	1.313
Rich Vs Poor^R^	-0.006	0.090	-0.070	0.943	0.993	0.823	1.197
**Age at first birth**							
<20years Vs ≥20 years^R^	0.071	0.100	0.740	0.457	1.074	0.888	1.298
**Antennal care visit**							
1–2 times Vs No^R^	0.063	0.100	0.650	0.517	1.065	0.879	1.290
3 and more times Vs No^R^	0.218	0.120	2.200	0.028	1.243	1.024	1.510
**Mode of delivery**							
Caesarian Vs Vaginal^R^	-1.196	0.030	-10.62	0.001	0.302	0.242	0.377

R:Reference Category

## Discussion

EIBF is one of the most effective practices for providing balanced nutrition for newborn babies, and to reduce the rate of child mortality and morbidity. The overall prevalence of the EIBF practice among Bangladeshi mothers as based on the BDHS survey taken in 2014, which was 51.4%, indicating some improvement when compared to the rate of 47% reported in the survey conducted in 2011 [23]. This rate was lower than that for the neighboring country of Nepal (66.4%) [28], as well as for other developing countries such as Ethiopia (83.7%) [14]. However, the rate was higher than other South Asian countries like India **(**21%**),** Pakistan (8.5%), and other developing countries like Nigeria (34.7%), Iran **(**32.2%), and South Sudan (48%) [32–36].

After removing the clustering effect of the dependent variable and control for the effect of other factors, it was noted that the mode of delivery was the most important predictor for EIBF among Bangladeshi women. Mothers who underwent caesarean sections were less likely to initiate early breastfeeding as compared to those who underwent vaginal delivery. Since most caesarean sections were performed under general anesthesia, breast feeding within the first hour after delivery would be technically difficult or impossible because of the time needed for recovery from general anesthesia, which may take a few hours. Even if the procedure were performed under a regional or spinal anesthesia, most operating theaters do not have facilities for breast-feeding in the recovery area. This finding has also been reported by other studies [14, 33].

Geographical location is another important predicting factor for EIBF. Mothers living in the Sylhet division were more likely to provide their breast milk within one hour after birth than mothers living in other divisions, the same finding was also observed in a previous Bangladeshi study [37]. This may be related to the customs and the culture of the local community. However, the mode of delivery may further contribute towards this finding since the Sylhet division has reported the highest rate of home delivery (76.6%), and lowest rate of caesarean sections in Bangladesh [23].

This study also demonstrated that mothers who lived in a rural environment were more likely to commence early breastfeeding as compared to mothers from an urban environment. This has also been reported in the previous study in Bangladesh [37]. Mode of delivery may also serve as one of the contributing factors in this study. It has been reported that 69.1% of mothers in the rural areas delivered at home, and that urban mothers were twice (38%) as likely as rural mothers (18%) to delivery by caesarean section [23]. The association between EIBF and the rural environment was not supported by a study among mothers in Nigeria [34]. The finding suggests that there may be other factors that influence breast-feeding behavior in that nation. It was also noted that mothers with a higher education status were less likely to practice EIBF compared to those who were not as educated. This observation has also been reported in a recent publication by Sakib et al [37]. This might also contribute towards a higher rate of EIBF in the rural area, since the level of education among mothers in these areas was generally low. It has been reported that 24.5% of mothers in urban areas of Bangladesh have higher education, while this was the case in only 10.4% of rural areas [23]. Delivery by caesarean section has gradually become more common among educated and working mothers in urban areas in Bangladesh [23]. The same tendency has been observed in Pakistan [38]. Interestingly, education has been shown to exert a positive influence towards the practice EIBF in Nepal [28], since educated mothers were probably more aware of the benefits of early breastfeeding in infants and for themselves. Therefore, despite our finding that showed a lower rate of EIBF among educated mothers, the possibility of educating expecting mothers on the benefits of EIBF in both the rural and urban areas may be a strategy to promote EIBF for the whole country.

This study showed that overweight or obese mothers were less likely to practice EIBF when compared to normal weight mothers in Bangladesh. A similar observation was reported by Krause et al [39] on overweight and obese mothers above the age 18 in USA. For mothers with a higher BMI, the indication for caesarean section may be due to a higher risk of cephalo-pelvic disproportion and relatively poor progress due to maternal fatigue [40]. In addition, these mothers in Bangladesh were more likely to be from wealthy families in the urban areas, where the facility for caesarean delivery was more readily available [23]. This study, moreover, revealed that mothers who underwent caesarean section delivery were less likely to provide their breast milk to their newborn within one hour after birth when compared to their counterparts. This is one of the reasons for the differences seen in the practice of EIBF between overweight/obese and normal weight mothers. These mothers should be the focus of education on the potential benefits of EIBF.

Finally, it was also observed that attendance of antenatal care (ANC) was found to be positively associated with EIBF among mothers. Mothers who attended an ANC clinic (3 or more times) were more likely to provide early breastfeeding to their newborn babies when compared to those who did not attend an ANC clinic at all. Based on these findings, the health authorities may want to improve the ANC clinic attendance among expectant mothers, and pay special attention to those who are overweight and obese. In most developing countries, early breastfeeding is an important subject of health education for women. Public health policy discouraging the advertisement on milk powder in ANC can help to promote EIBF, and this has been reported by the studies conducted in Ethiopia, Nepal and Nigeria [14, 28, 34]. Providing health education on the benefits of EIBF among women may further increase the practice of EIBF, and this may contribute towards reducing the maternal and child mortality rates.

The nationally representative data used in this study was collected from different levels of Bangladesh, and a cluster effect was found. One of the strengths of the present study was to remove this clustering effect to attain accurate risk factors for non-initial breastfeeding mothers in Bangladesh. This study has provided information for the authorities to target their resources and efforts to population groups who are less likely to practice EIBF. Medical and nursing staffs of ANC clinics can also try to encourage expectant mothers to increase the number of follow up visits, especially those who are overweight or obese. We hope that all these efforts to promote EIBF will eventually help Bangladesh to achieve the target of SDG 2030. However, it should be noted that there were some limitations with this study such as the information was derived from a secondary source, and one could not investigate some of the causal associations between certain exposures and the outcome variable. Although one has considered some of the socio-economic and demographic factors for investigating their effect on EIBF, other possible influences such as health facility, the desire of pregnancy, could not be analyzed. We are also aware of the risk of recall bias considering the nature of some of these questions, such as the child’s birth weight, which may not be recorded in any document.

## Conclusions

This study showed that almost half of all Bangladeshi mothers do not practice EIBF. Mothers from specific geographical regions like the Sylhet division, and those from rural areas were more likely to practice EIBF. Uneducated, healthy (normal weight) and vaginally delivered mothers were more likely to provide their initial breast milk to their newborns. It was also noted that mothers who attended ANCs more than 3 times were more likely to perform EIBF. Thus, providing information on the benefits of EIBF among educated women in urban areas may also be an effective way to promote EIBF, with a special focus on expecting mothers attending the ANC clinics.
